# Host anemone size as a determinant of social group size and structure in the orange clownfish (*Amphiprion percula*)

**DOI:** 10.7717/peerj.5841

**Published:** 2018-11-06

**Authors:** Juliette Chausson, Maya Srinivasan, Geoffrey P. Jones

**Affiliations:** 1 College of Science and Engineering, James Cook University, Townsville, QLD, Australia; 2 ARC Center of Excellence for Coral Reef Studies, James Cook University, Townsville, QLD, Australia

**Keywords:** Papua New Guinea, Social hierarchy, Coral reefs, Anemonefish, Depth, Patch size, Pomacentridae, *Amphiprion percula*, *Heteractis magnifica*

## Abstract

The size and structure of social groups of animals can be governed by a range of ecological factors and behavioral interactions. In small, highly site-attached coral reef fishes, group size is often constrained by the size of the habitat patch they are restricted to. However, group size may also be influenced by changes in abundance along important environmental gradients, such as depth or distance offshore. In addition, the body size and sex structure within social groups can be determined by the size of the habitat patch and the dominance relationships among group members. Here we examined the roles of ecological factors and behavioral interactions in governing group size and structure in the orange clownfish, *Amphiprion percula,* on inshore reefs in Kimbe Bay, Papua New Guinea. We quantified relationships between ecological variables (anemone size, depth, and distance from shore) and social group variables (group size, and total body length of the three largest individuals (ranks 1, 2, and 3)). Anemone size explained the greatest amount of variation in group variables, with strong, positive relationships between anemone surface area and group size, and total length of individuals ranked 1, 2, and 3. Group structure was also weakly correlated with increasing depth and distance from shore, most likely through the indirect effects of these environmental gradients on anemone size. Variation in group size and the lengths of ranks 2 and 3 were all closely related to the length of rank 1. Path analysis indicated that anemone size has a strong direct effect on the length of rank 1. In turn, the length of rank 1 directly affects the size of the subordinate individuals and indirectly affects the group size through its influence on subordinates. Hence, anemone size directly and indirectly controls social group size and structure in this space-limited fish species. It is also likely that anemonefish have feedback effects on anemone size, although this could not be differentiated in the path analysis.

## Introduction

Animals often form social groups to benefit from cooperation in finding food, avoiding predators, and reproducing (Wilson, 1975; Krebs & Davies, 1981; Alcock, 2001). There are numerous ecological factors that influence optimal group size, including variation in resource availability and predation risk (Cody, 1971; Hamilton, 1971; Caraco & Wolf, 1975; Caraco, Martindale & Whitlam, 1980; Markham et al., 2015). Group size can also be determined by the size and quality of the habitat patches occupied—the well-supported “ecological constraints” model (Crook, 1970; Chapman & Chapman, 2000; Smith & Chapman, 2007). As the distribution and abundance of most animals vary along key environmental gradients such as temperature, light, rainfall, and depth (Krebs, 1972; Ricklefs & Miller, 1999), group size in social animals is also likely to vary as a consequence. Behavioral interactions among group members can also influence group size and the reproductive status and body size of group members, with competing individuals evicted from groups or prevented from joining them (Wilson, 1975; Gilmour, DiBattista & Thomas, 2005; Wong et al., 2007; Fitzpatrick et al., 2008). Given the multitude of factors that can affect a group’s size and structure, the relative importance of different factors and the relationships among direct and indirect effects is not always known.

Many small, specialized, and highly site attached coral reef fishes form small social groups associated with discrete patches of habitat, such as corals, sponges, or anemones (Fautin & Allen, 1992; Munday, Jones & Caley, 1997; Wong, Munday & Jones, 2005; D’Aloia, Majoris & Buston, 2011). Individuals are confined to the habitat patch, which provides shelter from predators, access to specialized food resources, and breeding sites. Habitat patch size and quality are likely to represent ecological constraints on group size, with several studies demonstrating that group size can be correlated with the size of the habitat patch (Wong, Munday & Jones, 2005; Thompson, Munday & Jones, 2007; D’Aloia, Majoris & Buston, 2011; Noonan, Jones & Pratchett, 2012). Because such specialized species live in relatively small social groups, their behavior is frequently characterized by strong social hierarchies, with the dominant member of a group often aggressively regulating the status, size, sex, and reproductive status of subordinates (Fricke, 1979; Shapiro, 1981; Buston, 2003a; Hobbs, Munday & Jones, 2004). Dominant individuals may control group size by evicting subordinates and preventing recruitment to the habitat patch (Elliott, Elliott & Mariscal, 1995; Buston, 2003b). Subordinates may restrict their food intake to prevent potential conflict with dominant individuals and avoid eviction, thereby affecting their body size (Wong et al., 2008). However, the different roles of patch size and behavioral interactions in determining group size and structure are often unknown.

The distribution and abundance of coral reef fishes often varies along major environmental gradients on coral reefs, such as distance offshore (Williams, 1991; Gust, Choat & McCormick, 2001; Pittman & Brown, 2011; MacDonald, Bridge & Jones, 2016) and depth (Choat & Bellwood, 1985; McGehee, 1994; Srinivasan, 2003; Jankowski, Gardiner & Jones, 2015; Jankowski, Graham & Jones, 2015; MacDonald, Bridge & Jones, 2016; Smallhorn-West et al., 2017). Group size is also likely to vary along the same gradients, with larger groups located in areas of highest abundance. The morphology of habitat patches, such as corals, sponges, and anemones, may also vary along depth or turbidity gradients (D’Aloia, Majoris & Buston, 2011). Coral colony size can increase with depth in response to declining light intensity, with more foliose and open morphologies persisting in deeper habitats to maximize energy acquisition (Hoogenboom, Connolly & Anthony, 2008). Anemones also symbiotically associate with zooxanthellae and likely maximize surface area at greater depths to increase light capture for photosynthesis (Sebens & DeRiemer, 1977). However, the relative effects of distance offshore or depth on habitat patch size and group size and structure in specialized reef fish have not been assessed.

Anemonefishes represent a tractable model system for examining the interrelationships among habitat patch size, location, and the size and structure of social groups. They live in an obligate association with anemones, a small and discrete habitat where fish must derive all critical resources. Larger anemones are likely to provide more shelter, as well as an increased area for reproduction and laying eggs. While the specific species of the anemonefish and the host play an important role in determining the range of potential fish body lengths and group sizes (Fautin & Allen, 1992; Huebner et al., 2012), group size does appear to be positively related to anemone size in a number of species (Fricke, 1980; Fautin, 1992; Mitchell & Dill, 2005). The anemonefish simultaneously provides reciprocal benefits to the anemone, which may reinforce relationships between anemone size, and group size, and group structure. For example, anemonefish provide protection against anemone predators, such as butterflyfish, through aggressive behavior (Godwin & Fautin, 1992; Porat & Chadwick-Furman, 2004). Anemonefish have also been shown to transfer carbon and nitrogen, at least partially in the form of ammonia, to their host and the host’s symbiodinium (Cleveland, Verde & Lee, 2010). The transfer of ammonia from fish to host results in increased the zooxanthellae concentrations and tissue regeneration potential in anemones (Porat & Chadwick-Furman, 2005; Roopin & Chadwick, 2009). Nocturnal anemonefish movement aerates the anemone, potentially facilitating a range of benefits, including gas exchange and debris removal (Szczebak et al., 2013). Overall, anemones hosting anemonefish exhibit greater growth, more frequent asexual reproduction, and lower mortality than those without resident anemonefish (Holbrook & Schmitt, 2005; Frisch et al., 2016).

Anemonefishes are protandrous hermaphrodites, the largest individual (rank 1) in the group generally being the female and the second largest (rank 2) the breeding male, the latter undergoing sex change following the death of the female (Fricke & Fricke, 1977; Fricke, 1979, 1983; Buston & Elith, 2011). In anemonefish groups, reproduction is restricted to the two dominant individuals regardless of the group size. There is a strong dominance and size hierarchy between all members of the group, with non-breeders essentially in a queue for reproductive status (Buston, 2003a). The body size structure of groups may also be determined by anemone size (Mitchell & Dill, 2005), although Buston (2003a) determined that anemone diameter had no effect on the body lengths of subordinates in *Amphiprion percula*. Group size may be directly influenced by density dependent processes impacting the whole group or may be dictated by the size of the largest individual (Buston, 2003c; Mitchell & Dill, 2005). However, cause and effect can be difficult to assess when there is strong covariation between anemone size, group size, and body size. To date, few studies have examined the potential effects of both environmental and social control on relationships among group size and body size structure in clownfishes.

The aim of this study was to quantify relationships among anemone size, depth, distance offshore, group size, and body size structure in the orange clownfish, *A. percula* on inshore reefs in Kimbe Bay, Papua New Guinea. In addition, we conducted a path analysis to determine whether anemone size has a direct effect on group size or whether anemone size controls the size of the largest individual, who then dictates the size and structure of the social group. The following specific questions were addressed: (1) Are group size and structure positively related to anemone size? (2) Does anemone depth and/or distance offshore explain variation in group size and structure, and can this be attributed to spatial variation in anemone size? (3) Are the group size, and the size of rank 2 and rank 3 individuals related to the size of rank 1? (4) Does anemone size directly determine group size (and the size of subordinates) or is this mediated by the size of the rank 1 individual?

## Materials and Methods

### Study site and species

**Figure 1:**
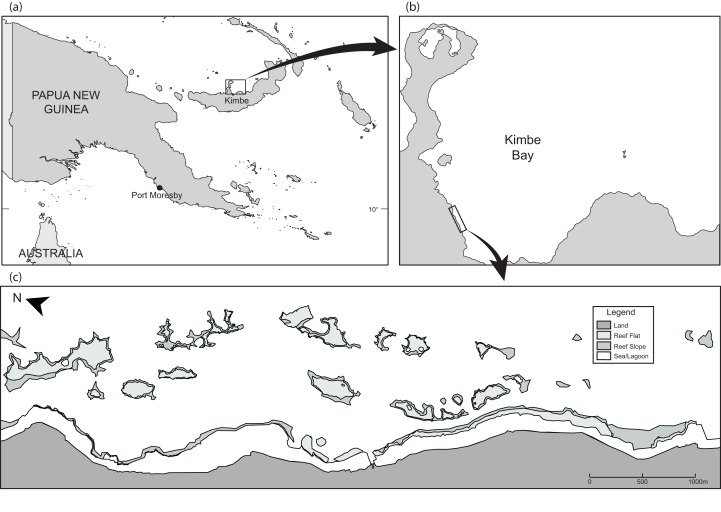
Maps showing (A and B) the location of Kimbe Bay and (C) the 19 inshore reefs.

This study was undertaken in April 2016 on the inshore coral reefs near the Mahonia Na Dari Research and Conservation Centre, near the town of Kimbe, in Kimbe Bay, Papua New Guinea (05°26′S, 150°52′E) (Figs. 1A and 1B). It focused on an area of the fringing reef approximately five km long as well as 19 discrete emergent reefs ranging a distance of 0.2–1.4 km from the coastline (Fig. 1C). Kimbe Bay experiences a distinct wet season from December to January, and a windy season from June to July, but there is little variation in temperature year-round (Srinivasan, 2003).

This study focused on the orange clownfish, *A. percula,* which is associated primarily with *Heteractis magnifica* and *Stichodactlya gigantea* anemones in this region, usually at depths shallower than 12 m (Almany et al., 2007; Planes, Jones & Thorrold, 2009). Due to the relatively low numbers of *S. gigantea* on coastal reefs, this study focused primarily on *A. percula* groups associated with *H. magnifica. A. percula* lives in small social groups with a strong dominance hierarchy, whereby individuals of sequential rank follow a fixed size ratio (Buston, 2003a, 2003b, 2003c). Reproduction in *A. percula* is also restricted to the two highest rank individuals: the highest rank generally being the female and the second highest rank generally being the male, if they have achieved the size required for sexual maturation (Buston & Elith, 2011). Recruitment to the group is restricted until the lowest ranked juvenile reaches a critical size (Elliott, Elliott & Mariscal, 1995; Buston, 2003b). Because this study did not incorporate gonad analyses, and the length of certain largest individuals were potentially below the length for reproductive maturity, individuals measured are referred to by their size ranking: rank 1 (the largest individual), rank 2 (the second largest individual), and rank 3 (the third largest individual).

The research in this study was conducted in compliance with the Queensland Animal Care and Protection Act 2001, and with the consent of the Mahonia Na Dari Research and Conservation Center, and the Local Marine Management Committee and traditional owners in Kilu.

### Field methods

The majority of the *A. percula* groups were located on the fringing reef and 19 adjacent reefs by systematic searches of all the reef area shallower than 20 m (Figs. 1B and 1C). The depth and GPS coordinates were recorded for all located anemones and *A. percula* groups. For each anemone, a photograph of the entire anemone, with calipers for scale, was taken to estimate the effective surface area. The number of *A. percula* in the anemone was recorded. All individuals in each social group were caught using hand nets, and the total length (TL) of each individual was measured using plastic calipers before releasing it back onto the host anemone.

### Environmental variables

All anemones were mapped using QGIS software, and distance from shore was calculated as the linear distance between each anemone and the closest point on the shore. Using Digital Earth Watch software, tentacle crown surface area was calculated from the anemone photographs (Dixon, McVay & Chadwick, 2017). This process involved virtual drawing along the contour of the anemone, including specific tentacles, to capture the full extent of the surface area. Anemones that appeared to have contracted tentacles and oral discs when photographed were not included in statistical analyses of anemone size, as measurements of surface area would have been underestimated.

### Group size and structure

Four variables were measured to quantify the social group structure of *A. percula*: (1) social group size, or the number of fish per anemone, (2) TL of rank 1 (the largest individual), (3) TL of rank 2 (the second largest individual), and (4) TL of rank 3 (the third largest individual). Only the lengths of the three largest individuals were assessed, because few *A. percula* groups contained more than three individuals per anemone in the area surveyed.

### Effects of environmental variables on group size and structure

Separate linear regressions between each environmental variable (anemone size, depth, and distance from shore) and each social structure variable (group size, TL of rank 1, TL of rank 2, and TL of rank 3) were fitted. The environmental variables were log transformed to ensure a linear relationship with social structure variables and to ensure the data conformed to the assumptions of linear regression. The potential relationships between anemone size and the other environmental variables (depth and distance offshore) were also assessed.

As there were significant correlations between environmental variables, correlation coefficients were calculated and compared against thresholds of 0.7–0.8, levels recommended for conducting regressions without multicollinearity posing an issue (Green, 1979; Menard, 2002). Subsequently, the variance inflation factor (VIF) was calculated using the equation: VIF*j* = 1/(1−*R*_*j*_^2^). The VIF was then compared against thresholds of 4–10, below which multicollinearity would not be a concern (Harrell, 2001; Guisan, Edwards & Hastie, 2002; Hair et al., 2006).

### Effect of rank 1 on social structure

The possible effects of the size of the rank 1 individual on group size and structure were analyzed using separate univariate regressions, with length of rank 1 as the independent variable, and the social group size, length of rank 2, and length of rank 3 as the dependent variable in each regression. The length of rank 1 was used as the social control factor, as previous studies have shown that female size (always the rank 1 individual) is a significant predictor of social group size and the size of subordinate individuals (Buston, 2003a; Mitchell & Dill, 2005).

### Path analysis

A path analysis was conducted using structural equation modelling to determine the likely pathways that link anemone size to social group size and structure. Path analysis imposes directional causation from one variable to the next and includes the magnitude of such effects. Relationships in nature are often not solely unidirectional and, as in the case of the relationship between anemone and anemonefish, may involve feedback loops where both members yield influence on each other. Nevertheless, path analysis allows exploration of the strength of interactions between many interacting factors in a potentially complex model (Streiner, 2005). Therefore, use of the path analysis in this study provides a useful platform for assessing the magnitude of both environmental and social influences on the group structure of anemonefish.

The path analysis in this study was conducted in R using the Lavaan package and devised using the results from the individual regressions as a starting point. The causation directions were informed both from previous literature on the hierarchy of *A. percula* group structure (Buston, 2003a) as well as a basic assumption that anemone size may be influenced by both depth and distance offshore. The fit of the model was evaluated by examining the *R*^2^ of the variables and comparing fit indices to accepted thresholds (e.g., a Comparative Fit Index of greater than 0.95 and a standardized root mean square residual below 0.08) (Browne & Cudeck, 1993; Hu & Bentler, 1999). Direct effects were determined by examining the path coefficients. Indirect effects were assessed by first determining the compound path and subsequently multiplying the coefficients of the component paths. The use of path analysis in this study is an a posteriori approach, fitting a model to a known data set for explanatory analysis, as opposed to creating a model prior to examining the data available.

## Results

### General patterns

A total of 98 *H. magnifica* with resident fish were mapped and sampled on the 19 inshore reefs. Six of these anemones were not included in statistical analyses of anemone size as they were contracted when photographed, and therefore measurements of surface area would have been underestimated. The distances from shore of the 92 anemones included in statistical analyses ranged from 185 to 1,400 m, and the depths occupied ranged from 3.3 to 19 m. The size of *A. percula* social groups ranged from two to five individuals per anemone.

### Are group size and structure positively related to anemone size?

**Figure 2:**
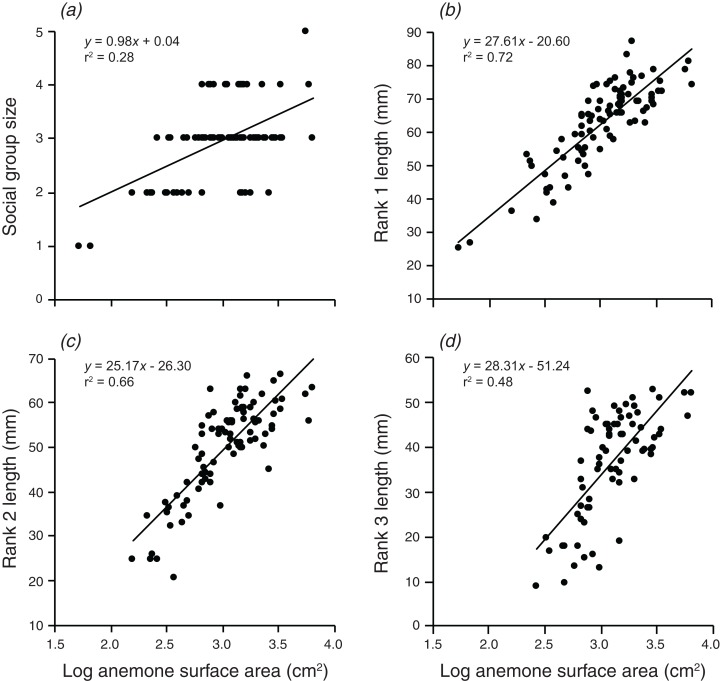
Relationships between log anemone surface area and (A) social group size, (B) rank 1 length, (C) rank 2 length, (D) rank 3 length. Lines indicate significant relationships.

Social group size, body length of rank 1, body length of rank 2, and body length of rank 3 all increased significantly with an increase in the log of anemone surface area (Fig. 2). Anemone size explained 28.3% of the variation in group size, 72.4% of the variation in rank 1 length, 65.5% of the variation in rank 2 length, and 48.25% of the variation in rank 3 length. All linear regressions were statistically significant (*F*_1,90_ = 35.7, *p* < 0.001; *F*_1,90_ = 236.1, *p* < 0.001; *F*_1,88_ = 167.7, *p* < 0.001; *F*_1,72_ = 67.14, *p* < 0.001).

### Does anemone depth or distance offshore explain variation in group size and structure, and can this be attributed to spatial variation in anemone size?

**Figure 3:**
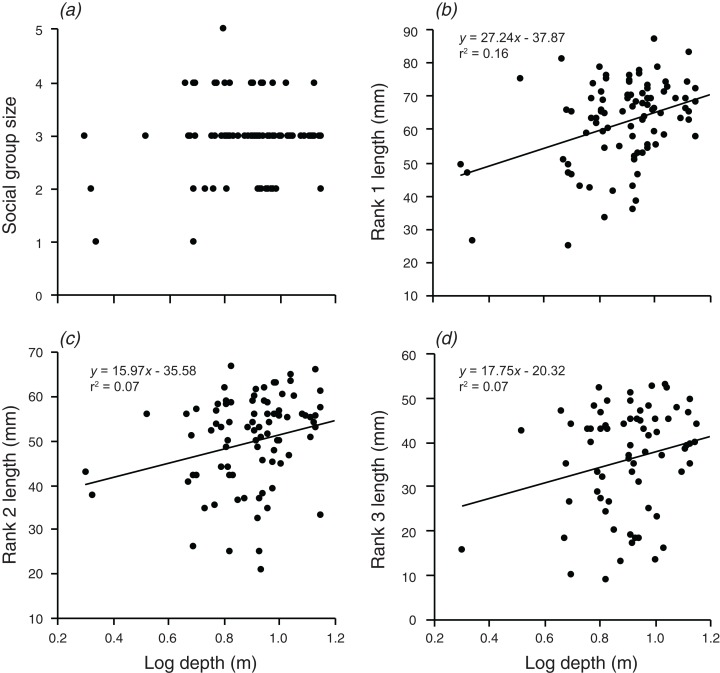
Relationships between log depth and (A) social group size, (B) rank 1 length, (C) rank 2 length, (D) rank 3 length. Lines indicate significant relationships.

There were significant, weak positive relationships between depth of the anemone and the body lengths of rank 1 rank 2, and rank 3 (*F*_1,90_ = 17.3, *p* < 0.001; *F*_1,88_ = 6.591, *p* = 0.012; *F*_1,72_ = 5.054, *p* = 0.028; Fig. 3). These relationships explained 16.1% of the variation in rank 1 body length, 7.0% of the variation in rank 2 body length, and 6.6% of the variation in rank 3 body length, respectively. There was no significant relationship between social group size and depth of the anemone (although there was a positive trend).

**Figure 4:**
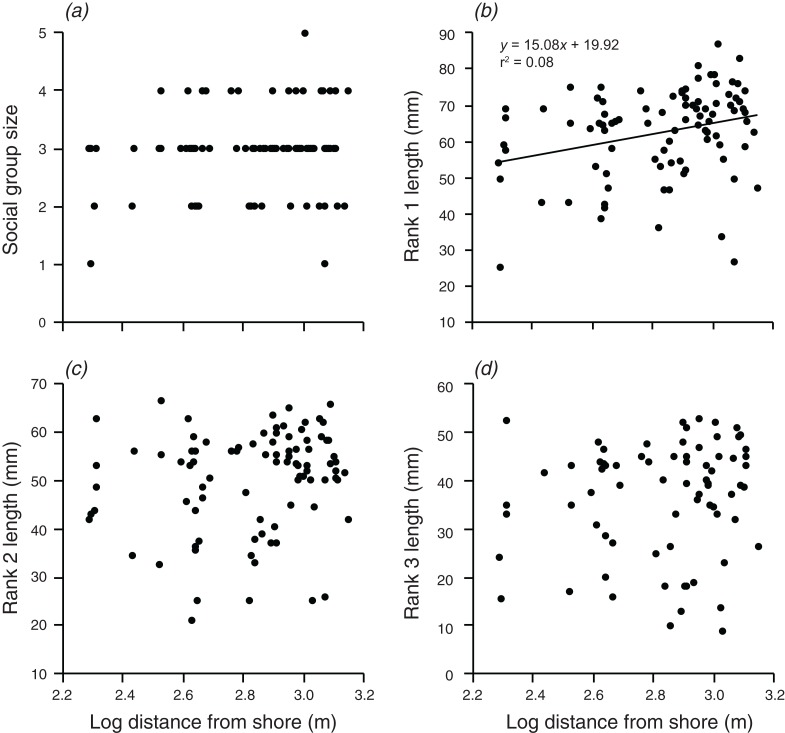
Relationships between log distance from shore and (A) social group size, (B) rank 1 length, (C) rank 2 length, (D) rank 3 length. Lines indicate significant relationships.

Distance from shore had no significant effect on group size, the body length of rank 2, or the body length of rank 3 (Fig. 4). However, there was a weak positive relationship between body length of rank 1 and the log of the distance from shore, which explained 8.2% of the variation in the length of rank 1 (*F*_1,90_ = 7.991, *p* = 0.006; Fig. 4).

**Figure 5:**
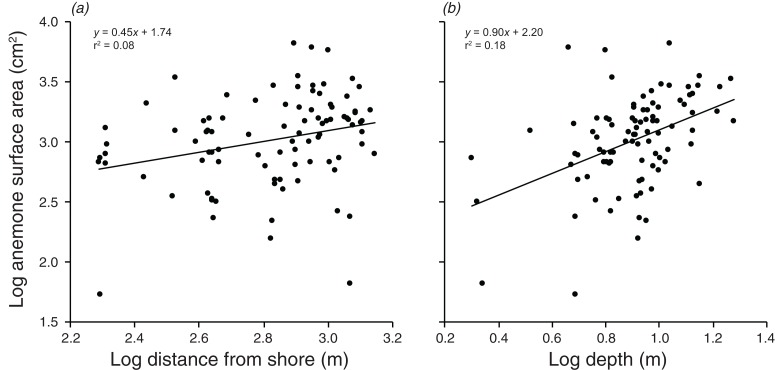
Relationships between log anemone surface area and (A) log distance from shore, and (B) log depth. Lines indicate significant relationships.

There were significant positive relationships between the log of the anemone surface area and the log of the distance from shore, and the log of the depth (*F*_1,90_ = 7.515, *p* = 0.007; *F*_1,90_ = 20.35, *p* < 0.001 values; Fig. 5). Depth and distance from shore were weakly correlated with each other (*r* = 0.22, *t*_2,90_ = 2.155, *p* = 0.034). A multiple regression with depth and distance from shore as independent variables explained 21.9% of the variation in anemone surface area (*F*_2,89_ =12.51, *p* < 0.001). The VIF factor was 1.28, indicating that the collinearity among the environmental factors did not limit the ability to distinguish among variables.

### Are the group size, and the size of the second and third largest individuals related to the size of the largest individual?

**Figure 6:**
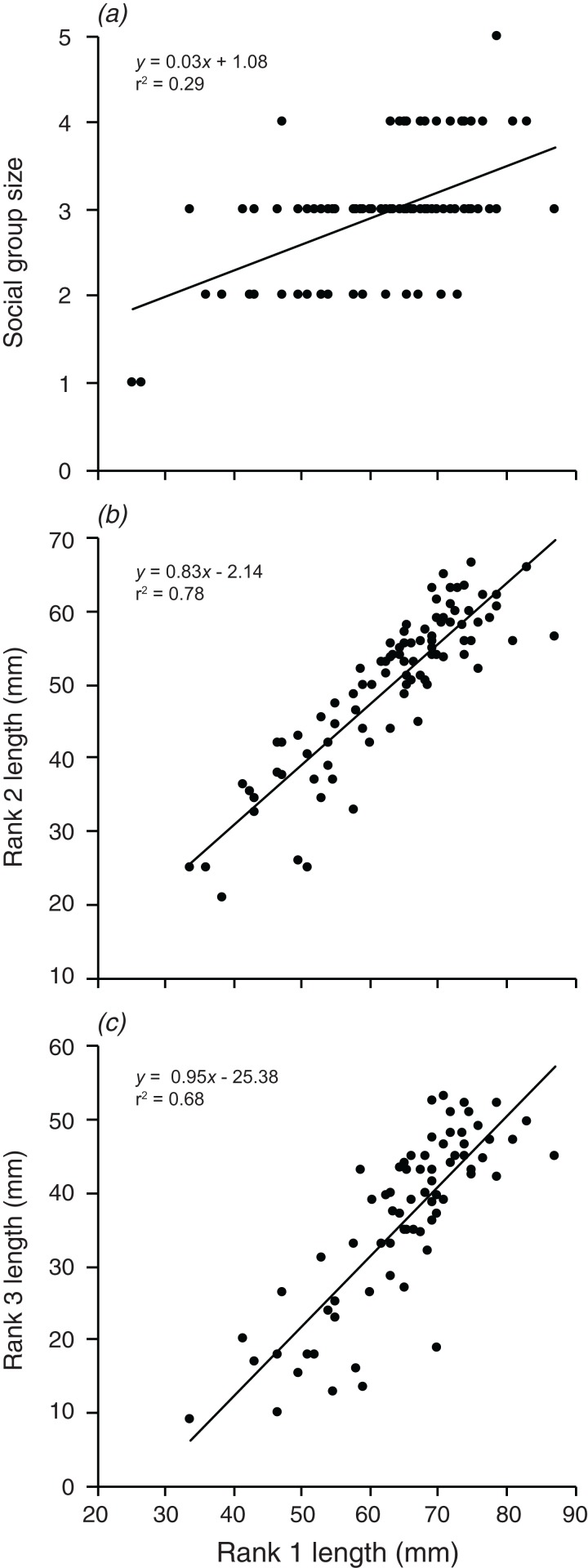
Relationships between the length of the rank 1 individual and (A) social group size, (B) rank 2 length, (C) rank 3 length. Lines indicate significant relationships.

Social group size, body length of rank 2, and body length of rank 3 each increased significantly with an increase in body length of rank 1 (*F*_1,90_ = 36.74, *p* < 0.001; *F*_1,88_ = 315.4, *p* < 0.001; *F*_1,72_ = 152.2, *p* < 0.001; Fig. 6). Rank 1 body length explained 29.0% of the variation in group size, 78.2% of the variation in rank 2 length, and 67.9% of the variation in the length of rank 3.

### Does anemone size directly determine group size or is this mediated by the size of the largest individual?

**Figure 7:**
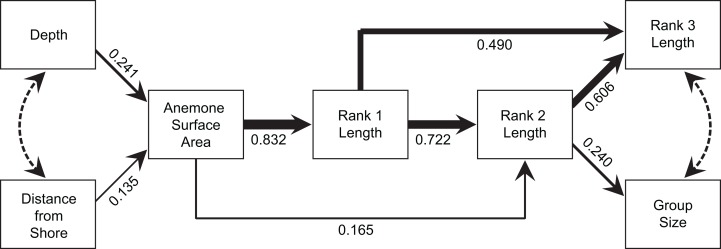
Path analysis showing the influence of environmental and social variables on social group size and structure. Direct effects are indicated by solid lines, with line thicknesses indicating the strength of the effect. Path coefficients, standardized versions of linear regression weights, are indicated below each solid line. Covariances are indicated by dotted lines.

The model produced by path analysis explained between 58% and 78% of the variation in the continuous group structure variables. The model fit the data well, with a Comparative Fit Index of 0.992, a Tucker-Lewis Index of 0.986, a root mean square error of approximation of 0.052, and a standardized root mean square residual of 0.051 (Fig. 7). Depth and distance from shore maintained weak, but direct, effects on anemone surface area. Anemone surface area had a strong direct effect on rank 1 body length, and rank 1 body length had strong, direct effects on the rank 2 body length and rank 3 body length. Through its effects on subordinate lengths, rank 1 maintained an indirect, yet important effect on social group size. Within this model, the direct effect of the anemone surface area on rank 1 body length was the strongest, direct interaction. However, anemone surface area also had positive, indirect effects on social group size and structure, via its effect on rank 1 body length (Fig. 7).

## Discussion

Overall, the results indicate that both environmental and social factors play important roles in determining group size and structure in *A. percula* associated with *H. magnifica* anemones in Papua New Guinea. Anemone surface area was clearly the most influential environmental variable, with larger social groups and larger individuals inhabiting larger anemones. Distance from shore and depth also had positive, indirect effects on group structure, most likely through their positive effects on anemone surface area, whereby anemones were larger at greater depths and distances from shore. Social factors also played an important role in regulating social group structure, with rank 1 positively affecting social group size, the body length of rank 2, and the body length of rank 3. Examination of these combined effects through path analysis demonstrated that anemone surface area directly affected the structure of rank 1 and rank 2 body lengths, and influenced rank 3 length and group size through the direct effects on the two highest ranking individuals.

### Critical importance of anemone size

Our results confirm those of previous studies showing that anemone size has a major influence on the size and structure of social groups of anemonefishes (Fricke, 1980; Fautin, 1992; Mitchell & Dill, 2005; Dixon, McVay & Chadwick, 2017). Anemone size was a good predictor of group size, and the lengths of rank 1, rank 2, and rank 3 individuals present on the anemone. The strong relationship between anemone size and rank 1 length is consistent with previous work (Elliott & Mariscal, 2001; Mitchell & Dill, 2005). Buston (2003a) did not find this relationship for *A. percula* in Madang, Papua New Guinea, suggesting some spatial variation in this phenomenon. It is important to note that the strength of this interaction is likely to be reinforced by reciprocal benefits that anemonefish bestow on their hosts, including defense (Porat & Chadwick-Furman, 2004; Godwin & Fautin, 1992) and provision of nutrients (Porat & Chadwick-Furman, 2005; Cleveland, Verde & Lee, 2010; Roopin & Chadwick, 2009; Roopin et al., 2011).

### Role of depth and distance offshore

Although the regression analysis suggested depth did not have a direct effect on social group size, deeper anemones certainly supported larger rank 1 and rank 2 individuals. Because the highest rank individuals tend to be the breeding pair, and fecundity increases with body size (Saenz-Agudelo et al., 2015), this relationship may have important consequences for reproductive potential. The depth effect is most likely related to the reduction in light with increasing depth. It is known that anemones have diel contraction patterns in response to the availability of light, with tentacles that have high densities of zooxanthellae responding to levels of light intensity (Sebens & DeRiemer, 1977). Anemones in shallow areas may retain retracted tentacles due to the presence of potentially high levels of photosynthetically active radiation, which can adversely affect the anemone and its *Symbiodinium* by inhibiting photosynthesis (Mass et al., 2010). Anemones at greater depths may conversely expand their oral disc to capture more light for photosynthesis, as light intensity is reduced at greater depths. However, anemones have also been shown to increase the concentration of their *Symbiodinium* at depth as a way to increase their photosynthetic capability (Dixon et al., 2014), and thus an alternative explanation is that anemones might actually be larger at greater depths, due to reduced wave action, increased nutrient availability, or other environmental parameters (Sebens, 1982). Either way, the outcome is a greater surface area available for *A. percula* on deeper anemones.

Distance from shore also had a weak positive effect on rank 1 length, again, most likely as a result of the effect of distance offshore on anemone surface area. Anemones that were further offshore tended to be slightly larger. The presence of smaller anemones closer to shore may be due to the likely higher levels of sedimentation near the fringing reefs. Increased sediment has previously been linked to a reduction in photosynthetic ability and an increase in tissue bleaching in anemones (Goreau, 1964; Rogers, 1990). Anemones produce mucus to remove sediment (Patton, 1979), which may be energetically costly and result in reduced growth in waters with higher sediment loads. Higher levels of sediment could also cause the anemone to contract to prevent accumulation of sediment. Conditions further offshore may also foster more favorable environments in terms of nutrient availability, current patterns, and, more broadly, a reduction in anthropogenic stressors (Smith et al., 2008). One or both of these mechanisms would lead to a decrease in anemone surface area with proximity to the shore and a smaller area for *A. percula* to inhabit.

### Role of the largest individual

The size of the rank 1 individual (in most cases—the female) exhibited strong relationships with rank 2 body size, rank 3 body size, and the group size. This is likely the outcome of the strong social hierarchy in anemonefish groups, and our results are consistent with other work on anemonefishes (Fricke & Fricke, 1977; Fricke, 1979, 1983; Godwin, 1994; Buston, 2003a; Iwata et al., 2008). Mitchell & Dill (2005) concluded that social group size in the sister species, *A. ocellaris,* is predominantly controlled by the dominant female (rank 1), based on the effect of female size on the size structure of the group. Buston (2003a) has previously established the importance of social control on individual body lengths in *A. percula*, with a fixed size difference between fishes that are sequentially ranked in the social hierarchy. With such fixed size ratios, the larger the rank 1 individual, the more individuals can be accommodated in the group. The mechanism controlling group size in anemonefish is likely forcible eviction or competitive exclusion (Buston, 2003b), leading to excluded fish finding refuge in potentially less preferable hosts (Huebner et al., 2012). Lower social rank has been associated with lower survivorship in *A. percula*, likely due to competitive eviction by higher ranked individuals (Buston, 2003c). Subordinate individuals may in turn be reducing their food intake to avoid exceeding size thresholds that could lead to eviction, as observed in other space-limited fishes (Wong et al., 2008). Hence, the size of the group and its structure may be determined by complex top-down and bottom-up interactions between competing individuals in the social hierarchy, including the species of host anemone.

### Distinguishing direct and indirect effects

It is very difficult to tease apart the relative importance of environmental factors (e.g., anemone size) and social factors (e.g., size of individuals by rank) in determining social structure variables, such as group size or the size of the subordinate fishes, since all the variables are correlated. Anemone size may directly influence group size through strict space limitation and associated density-dependent processes, or indirectly via the effect of anemone size on rank 1 size (Mitchell & Dill, 2005). Buston (2003a) concluded that the dominant female’s body length (rank 1 length) controlled the body lengths of the subordinates, because anemone diameter did not have a significant effect. Mitchell & Dill (2005) reached similar conclusions on the basis of stronger relationships with female size, compared with anemone size. Our path analysis strongly suggested that both direct and indirect effects are involved. Anemone surface area had a strong direct effect on rank 1 size and a slightly weaker effect on rank 2 size. The size of rank 1, in turn had a strong direct effect on the sizes of rank 2 and rank 3, while maintaining a substantial indirect effect on social group size through its effects on subordinates. Hence, anemone area had considerable indirect effects on all of the variables governing group size and structure. Further, the total effect (direct + indirect) of anemone size on rank 2 size was comparable to the direct effect of rank 1. The path analysis also demonstrated that the environmental variables (distance from shore, depth) have indirect effects on all four group structure variables through their influence on anemone surface area, although the effect of depth is arguably greater than that of distance from the coastline.

### Future directions

While the path analysis helps resolve the likely cause-effect links and direct vs indirect relationships, ultimately this complexity will need to be resolved by experimentation. Experiments have previously demonstrated that anemonefish provide important benefits to their hosts through a multitude of mechanisms. While Godwin & Fautin (1992) demonstrated the importance of *A. percula* for protection of anemones, other potential benefits, such as nutrient transfer and aeration, have yet to be tested for this species and need be resolved. Observation of the behavior of different individuals in such experimental designs may also aid in revealing the exact mechanisms responsible for control of group size and structure.

Further investigation is needed to understand the reasons underlying the effects of depth and distance offshore on social group structure. Variations in depth are coupled with variations in light intensity, temperature, and pressure (Levinton, 1982). Similarly, distance from the coastline often varies with other factors such as nutrient concentration, sediment runoff, and anthropogenic impacts (Fabricius, 2005; Wenger et al., 2015). It would be tractable to experimentally manipulate other environmental variables, such as light intensity and temperature, to fully understand how each variable influences the host anemones, and consequently, the structure of *A. percula* social groups.

There is increasing evidence that shallow water anemones experience severe bleaching after warm water events (Saenz-Agudelo et al., 2011; Hobbs et al., 2013). Bleaching not only reduced size and abundance of anemones (Hobbs et al., 2013), but further resulted in lower egg production and recruitment of anemone fish (Saenz-Agudelo et al., 2011). Higher temperatures have also been linked to breeding disruption, egg degradation, and possible connectivity among anemonefish populations (Munday et al., 2008, 2009). These effects are likely to have flow-on effects on the structure of clownfish social groups. Further attention should be directed to evaluating ongoing effects of climate change on the ecology and behavior of fishes that are so closely linked to the quality of their habitat.

## Conclusions

This study suggests that the surface area of the host anemone and the length of the largest individual are two critical factors defining the social group size and structure of *A. percula.* Variation in group structure along spatial gradients such as depth or distance from the coast may also be explained by the effects of these gradients on host anemone size. These results highlight the critical roles of habitat patch size and social hierarchy in governing breeding population size and access to limited resources in these highly specialized fishes. Additional experimental work is needed to fully resolve the direct and indirect effects of anemone size and the size of the largest individual on group size and structure, and how the behavior of this species will be shaped by increasing stresses on the anemone habitat.

## Supplemental Information

10.7717/peerj.5841/supp-1 Supplemental Information 1 Raw data collected during field sampling. Transformations applied for analysis are also included in the columns following each variable.

10.7717/peerj.5841/supp-2 Supplemental Information 2 Code for Path Analysis.

## Additional Information and Declarations

### Field Study Permissions

The following information was supplied relating to field study approvals (i.e., approving body and any reference numbers): This research was undertaken under a research visa issue by the government of Papua New Guinea (see scan attached for “Researcher/Academic” VISA for Geoffrey Jones from the Papua New Guinea Government). Verbal permissions were granted by: Mrs. Cecilie Benjamin (Chair of the Board, Mahonia Na Dari Research and Conservation Centre, Kilu) and Mr. Thomas Koi, (Village Elder and representative of the Local Marine Management Committee, Kilu).

### Data Availability

The following information was supplied regarding data availability: Raw data is provided in the Supplemental Files.
