# Supplementary material for: Host anemone size as a determinant of social group size and structure in the orange clownfish (Amphiprion percula)
Source: PeerJ. 2018 Nov 6;6:e5841. doi: 10.7717/peerj.5841 (PMC6225843; doi:10.7717/peerj.5841)
Supplement: Supplemental Information 2 [file peerj-06-5841-s002.docx]

**Path Analysis Code (Lavaan Package)**

Please note that the variables were standardized prior to entering into Lavaan (see data sheet for standardized values).

**Variable names:**

- **Log.SA.St** – Log(anemone surface area) standardized
- **Log.Depth.St** – Log(depth) standardized
- **Log.Dist.St** – Log(distance) standardized
- **Group.Size.St** – Group size standardized
- **Idv.1.St** – Idv 1 TL standardized
- **Idv.2.St** – Idv 2 TL standardized
- **SAGL.St** - Subadult summed TL standardized

**Code:**

*>* library(lavaan)

> Chausson_et_al_Data <- read.csv("~/Documents/Chausson_et_al_Data.csv")

> View(Chausson_et_al_Data)

> Model.Apercula <- '

Log.SA.St ~ Log.Depth.St + Log.Dist.St

Idv.1.St ~ Log.SA.St

Idv.2.St ~ Idv.1.St + Log.SA.St

SAGL.St ~ Idv.1.St + Idv.2.St

Group.Size.St ~ Idv.2.St'

> fit <- sem (Model.Apercula, data = Chausson_et_al_Data)

> summary(fit, fit.measures = TRUE)

> model.est <- sem (Model.Apercula, data = Chausson_et_al_Data)

> parameterEstimates(model.est)

> summary(model.est, standardized = TRUE, rsq = TRUE)
